# Coordination of protection and operation in micro-grids with inverter based distributed generation using SAOA

**DOI:** 10.1371/journal.pone.0311584

**Published:** 2024-10-10

**Authors:** Mohammad Hassan Tanha, Majid Gandomkar, Javad Nikoukar

**Affiliations:** Department of Electrical Engineering, College of Engineering Technology, Saveh Branch, Islamic Azad University, Saveh, Iran; Siksha O Anusandhan University Institute of Technical Education and Research, INDIA

## Abstract

In the proposed protection coordination scheme, the depreciation of the operation time of the entire relay in the primary and backup protection modes for all possible fault locations is considered as the objective function. The limitations of this problem include the equations for calculating the operation time of the relays in both forward and reverse directions, the limitation of the coordination time interval, the limitation of the setting parameters of the proposed relays, the restriction of the size of the reactance that limits the fault current, and the limitation of the standing time of distributed generation per small signal fault. The operation time of the relays depends on the short circuit current passing through them, so it is necessary to calculate the network variables before the fault occurs. For this purpose, optimal daily power distribution should be used in the micro-grid, because micro-grids consist of storage and renewable resources. The proposed plan includes the uncertainties of consumption and generation capacity of renewable resources. Then, to achieve a reliable answer with a low standard deviation, the refrigeration optimization algorithm is used to solve the proposed problem. Finally, the proposed design is implemented on the standard test system in the MATLAB software, and then the capabilities of the proposed design are examined.

## 1. Introduction

In interconnected electricity systems, due to economic savings, electric energy is generated centrally and by large power plants. In this situation, the cost of transmission and distribution of electrical energy increases significantly. Therefore, central production by large power plants often causes an increase in the cost of transmission and distribution, acute environmental issues, technological changes, and various impractical legislations. Therefore, factors such as environmental pollution, the problems of building new transmission lines and progress in the field of economic construction of small-scale generation units have increased the use of these units under the name of distributed generation (DG), which are mainly connected to distribution networks or micro-grids (MGs).

Another issue that should be considered is network protection, which is a basic necessity in designing electricity distribution networks or MGs against various faults. A proper protection plan should be able to clear the fault from the network as soon as possible by isolating the smallest possible part of the network. Protection coordination between the protection equipment in the distribution network is for the purpose that the main protection removes the fault from the network in the shortest time and in case of its failure, the backup protection comes into action and clears the fault.

Distribution networks or micro-grids are often operated radially and have one-way load distribution from the main substation to the loads. In these networks, overcurrent fuses and relays are used for overcurrent protection. The passage of an electric power line will make it easier to coordinate between protective equipment. However, by connecting DG to distribution networks for local energy supply to subscribers, the protection plan in distribution systems loses its effectiveness. With the connection of DGs to the distribution network, load distribution undergoes fundamental changes and the range of short-circuit current increases and its direction also goes out of one-way mode. The change in the short-circuit current of the network depends on the type, installation location and capacity of DGs.

References [1–4] first defined the sources of DG and then stated the reasons for using and replacing these sources instead of traditional power plants from the point of view of economic and environmental benefits. References [5, 6] have also dealt with the proper utilization of distributed renewable production resources in MGs.

References [7–9] mention some of the risks and dilemmas of using DGs along with their benefits. One of the most important of these problems is removing the network from the radial mode and disrupting the protection coordination.

References [10, 11] have discussed the impact of DGs on the short circuit level and as a result, the coordination of protective equipment based on the size and installation location of DGs. In the following, the effect of the type of DG on the level of short circuit is described to a great extent.

References [12–14] have expressed the complexities of protection coordination and calculation of short-circuit current in the presence of DGs. After that, methods are presented to reduce these complications. In such problems, because checking all cases is very time-consuming, the use of meta-heuristic optimization algorithms is suggested.

In [15, 16], by defining an optimization problem for protection coordination and solving it using meta-heuristic algorithms, the main parameters in setting relays have been found.

Also, the use of various types of Fault Current Limiters (FCL), including resistive and self-contained, has been evaluated in [15]. Other devices of the power system have also been reviewed in [17–19] and the useful life of their use has been increased.

The use of Dual Setting Directional Overcurrent Relays (DS/DOCR) can greatly reduce the coordination time interval [20]. It can also reduce the overall operation time of primary and backup relays by maintaining coordination.

Adaptive protection coordination for MGs using an improved optimization technique for user-defined DOCR relays is presented in [21]. Also, a review of optimization techniques for relay coordination considering adaptive MG designs is given in [22].

Adaptive harmonic-based protection coordination for inverter-based isolated MGs considering N-1 probability is investigated in [23]. A new protection scheme for MG feeders using inverter-based sources is also given in [24]. Modelling and study of the limiting effect of dynamic reactive current in distribution network inverters has also been analyzed in [25].

An innovative approach that proposes the use of Battery Electric Locomotives (BEL) as mobile energy storage devices is proposed according to [26]. BEL carries detachable battery railcars with increased storage capacity that provide a flexible and widespread energy source. Based on this, an uncertainty-aware optimization model is proposed that integrates the operation of power and railway systems in general. The proposed model is formulated as a mixed- integer nonlinear stochastic programming (SMINLP) that incorporates uncertainty through joint probabilistic constraints (JPCs). Also, in [27], a second-order converter with high gain and high efficiency is proposed. The proposed topology takes advantage of the good characteristics of the boost converter. Continuous input current, in addition to its low ripple, is one of the inherent characteristics of the proposed converter. In addition, the proposed converter performs switching with two switches on the bottom side, which simplifies its drive. The proposed converter is suitable for PV applications due to the mentioned features.

A two-way on-board charger used in electric vehicles is reviewed in [28]. The charger consists of a front-end hybrid PFC/inverter using a SEPIC/Zeta converter followed by an isolated symmetric full-bridge CLLC converter. SEPIC PFC maintains the DC link voltage in charging mode using battery pack voltage feedback. As a result, by switching the CLLC converter near the resonant frequency, it charges the appropriate voltage of the battery. Also, today, various types of PFCs are widely used. The main problem with these converters is that they cannot provide a wide output voltage range. Therefore, in [29], a hybrid PFC-Inverter is presented for two-way electric vehicle charging applications.

Reference [30] presents a single-level stochastic optimization framework for planning and partitioning a distribution system including multiple MGs (MMGs). The main objective is to minimize the total system cost including investment, operation, total loss and reliability costs of the distribution network. The proposed model considers the views of MG owners and distribution system operators simultaneously. Also, in [31], robust single-level methods for partitioning and scheduling Active Distribution Network (ADN) to multiple MGs are presented. According to the intended objective, the objective function of the model is to minimize the investment costs for the installation of distributed generation (DGs) capacity and switches, the activity of responsive loads based on the forecast of non-DG, losses and risk.

The rest of this paper is constructed as follows. In the second part, the solution procedure includes DG penetration determination, optimum protection coordination, and then SDGS effect on operation and protection will be expressed. In the third part, Simulated Annealing Optimization Algorithm (SAOA) will be introduced. In the fourth section, the results of maximum penetration capacity and DG effect on operation will be stated and discussed. Finally, the conclusion of the article will be examined.

## 2. Solution procedure

The presence of DGs can cause the unwanted operation of the protection system, the failure of protection equipment (protection failure), the creation of an uncontrolled island network, asynchronous reconnection and the reduction of the safety of the distribution network and MG.

The distribution network can host the installation of DGs if the protection solutions for their presence in the network are created. The potential of MG hosting can be enhanced by installing new protective equipment. Overcurrent relays are common protection equipment used in distribution networks. The use of these relays in the distribution network with the presence of DGs can be proposed as a protection plan in a necessary matter for accessibility and practicality. The optimum coordination between over-current relays is to meet the protection requirements of distribution networks and MGs.

In the conventional protection coordination strategy, TDS and I_P_ of the relays are optimally adjusted during the optimization process, and other coefficients such as A and B are fixed values. One of the features of numerical overcurrent relays is the possibility of defining the desired time-current characteristic curve coefficients by the user. In this strategy, in addition to TDS and I_P_, coefficients A and B are also considered as continuous variable settings that will cause different TCCs. Therefore, more flexibility is obtained and the total time of operation of the relays is reduced.

One of the major disadvantages of distributed generation resources is the direct impact on the protection of distribution networks so in some cases, protection plans lose their performance and coordination when synchronous DG (SDG) units are present. Therefore, an optimal amount of SDG capacity should be placed in the network so that while maintaining the protection coordination, the amount of SDG capacity in the network is at its maximum value. The main objective function includes the following:

- In the first stage, minimizing the overall operation time of protective relays,
- In the second step, find the maximum penetration capacity of SDG according to the Z_th_ of the network with the condition of maintaining the protection coordination
- In the third step, finding the maximum penetration capacity considering the limitations of losses, voltage profile, protection coordination and cost

**Fig 1:**
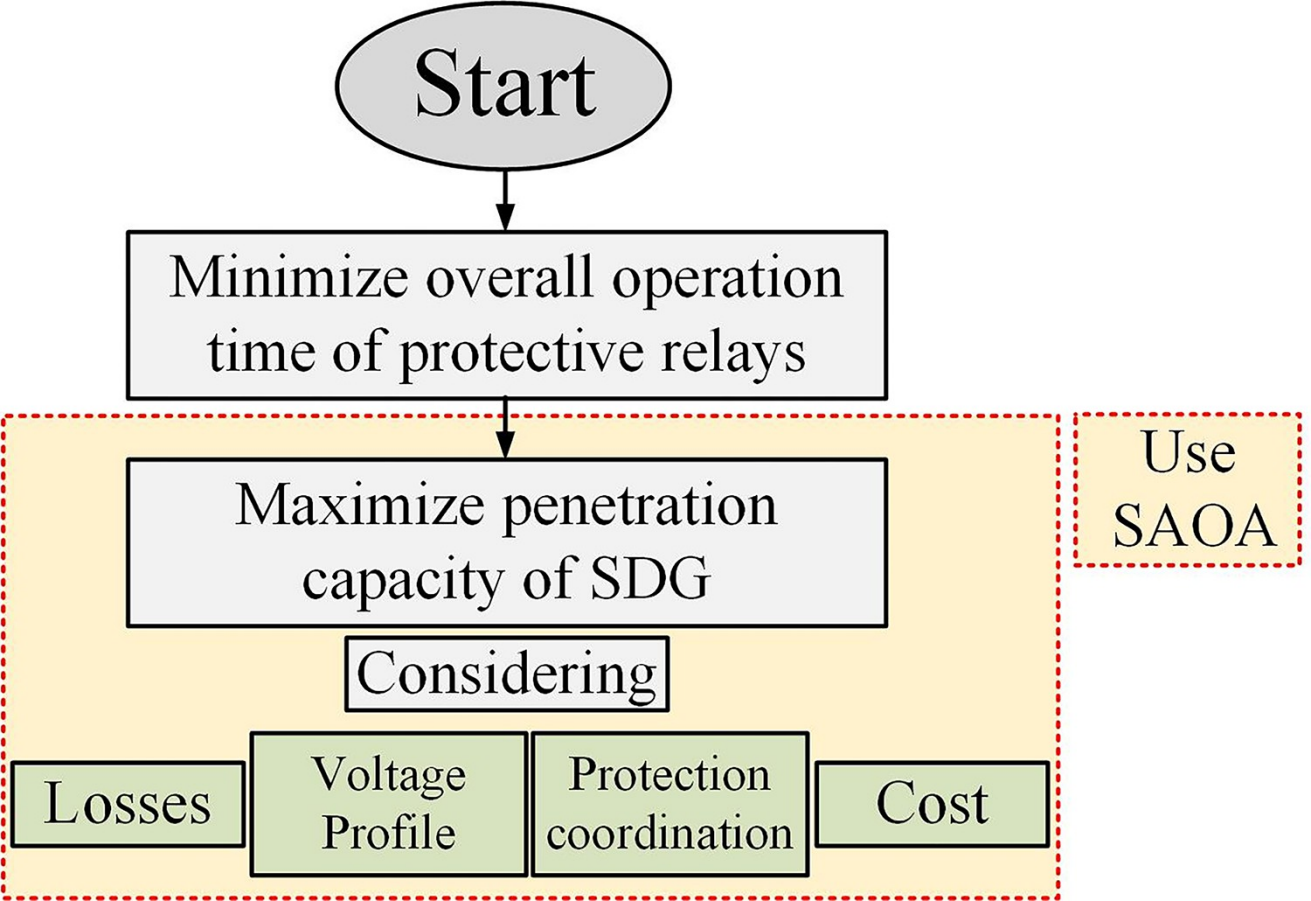
Flowchart of overall solution procedure.

Fig 1 shoes the overall solution procedure of this paper. In the following, each section will be explained.

### 2.1. DG penetration determination in the network

In protection coordination, the objective function is generally equal to the minimization of the operation time of the relays, which is proportional to the Eq (1).


$$t=TDS\frac{A}{{\left(\frac{I_{SC}}{I_{P}}\right)}^{B}-1}$$ (1)

In this regard, we have, t is the relay operation time, A is a constant parameter which is equal to 0.14, B is a constant parameter which is equal to 0.02, I_P_ pick-up current or excitation, TDS is a time setting parameter (Time-dial setting) and ISC is short circuit current.

In Eq (1), the expressions I_P_ and TDS are relay setting parameters, which in the protection coordination problem, these parameters are determined and calculated for each relay.

Next, two-stage optimization and finding the protection coordination index (PCI) will be discussed. Using the outputs obtained from the first phase of optimization, i.e. I_P_ and TDS, this article tries to keep these optimal values constant by introducing DGs.

The presence of DGs in the network causes that when a fault occurs, the fault current is provided through these sources in addition to the national network, and as a result, the short circuit current increases by injecting current from these sources at the fault location. It is obvious that the larger the DG impedance (Z_DG_) is, or the further the SDG installation location is from the fault location, the contribution of DG to the fault current decreases.

In the second stage, the increase in the capacity of SDGs in the distribution network and its effects on protection coordination are investigated. The goal at this stage is to set the maximum DG capacity, but the amount of injected short-circuit current caused by synchronous sources depends on the capacity, its location, its impedance and the fault point. If S_k_^SDG^ is considered as the capacity of SDG installed in bus k in terms of (MVA), the penetration rate of SDG is obtained by Eq (2) [32, 33].

$$Maximize\;P=\sum_{k\in K}S_{k}^{SDG}$$ (2)

where P is the total capacity of SDGs in terms of MVA, k is the bus counter, K is the total number of buses and S_k_^SDG^ is the capacity of the SDG that can be installed in bus k in terms of MVA. The total P value is obtained through optimization. The injection fault current of SDGs depends on its capacitance and impedance. Eq (3) expresses this value for the permanent injection fault current mode of SDGs [34].


ISCDG=f(SDG,ZDG)=(SDG3×VDG)ZDG (3)

Placing SDG in a specific bus, while changing the structure of the network, also affects the Z_th_ matrix of the network. With the addition of SDG to the network, there is no change in the dimensions of the impedance matrix of the network and only the values of the matrix are changed due to the impedance of the SDG. The current seen by the relay at the fault point can be expressed as Eq (4), which is the same value as the fault current in PSM [32].

$$I_{SCk}=f(S_{k}^{DG},Z_{sys})=\frac{V}{Z_{k}}=\frac{V}{\left(Y_{kk}+\frac{1}{Z_{k}^{DG}}\right)}$$ (4)

where I_SCk_ is the short-circuit current of bus k from the point of view of the relay in the presence of SDG, S_k_^SDG^ is the capacity of the SDG installed in bus k, V is the load distribution voltage in the presence of SDG, Z_k_ is the diagonal element number k of the Z_th_ matrix of the equivalent network in the presence of SDG, Y_kk_ is the equivalent admittance related to the buses where the SDG is placed and S_k_^SDG^ is the impedance of the SDG connected to bus k.

The impedance value of SDG decreases with increasing capacity. The constraint of the capacity of SDGs is in Eq (5) [32].


$$0\leq S_{k}^{DG}\leq S_{K}^{DG},\;\forall k$$ (5)

By placing a source with different capacities at one point of the network and creating a fault, its effect on the impedance of the network and the fault current level can be observed.

In Eq (6), the influence of SDGs is expressed as power changes (ΔP) in terms of CTI changes (ΔCTI) as the Protection Coordination Index (PCI), which has only calculated the impact of resources in terms of flow [32, 33]. The negative sign is to neutralize the effect of CTI changes due to the decrease from 0.3 seconds to 0.25 seconds.

$$PCI=-\frac{\Delta P}{\Delta CTI}$$ (6)

where ΔP is the change in the power of distributed production and ΔCTI is the change related to CTI.

The PCI index is determined as the rate of change of the maximum SDG penetration level according to the rate of change of CTI, and its unit is megavolt ampere per second. Higher PCI values at specific locations indicate that higher levels of SDG penetration will result in less impact on the protection coordination between relays (or in other words small changes in CTI) and this means that the bus is more sensitive to changes CTI is less sensitive.

**Fig 2:**
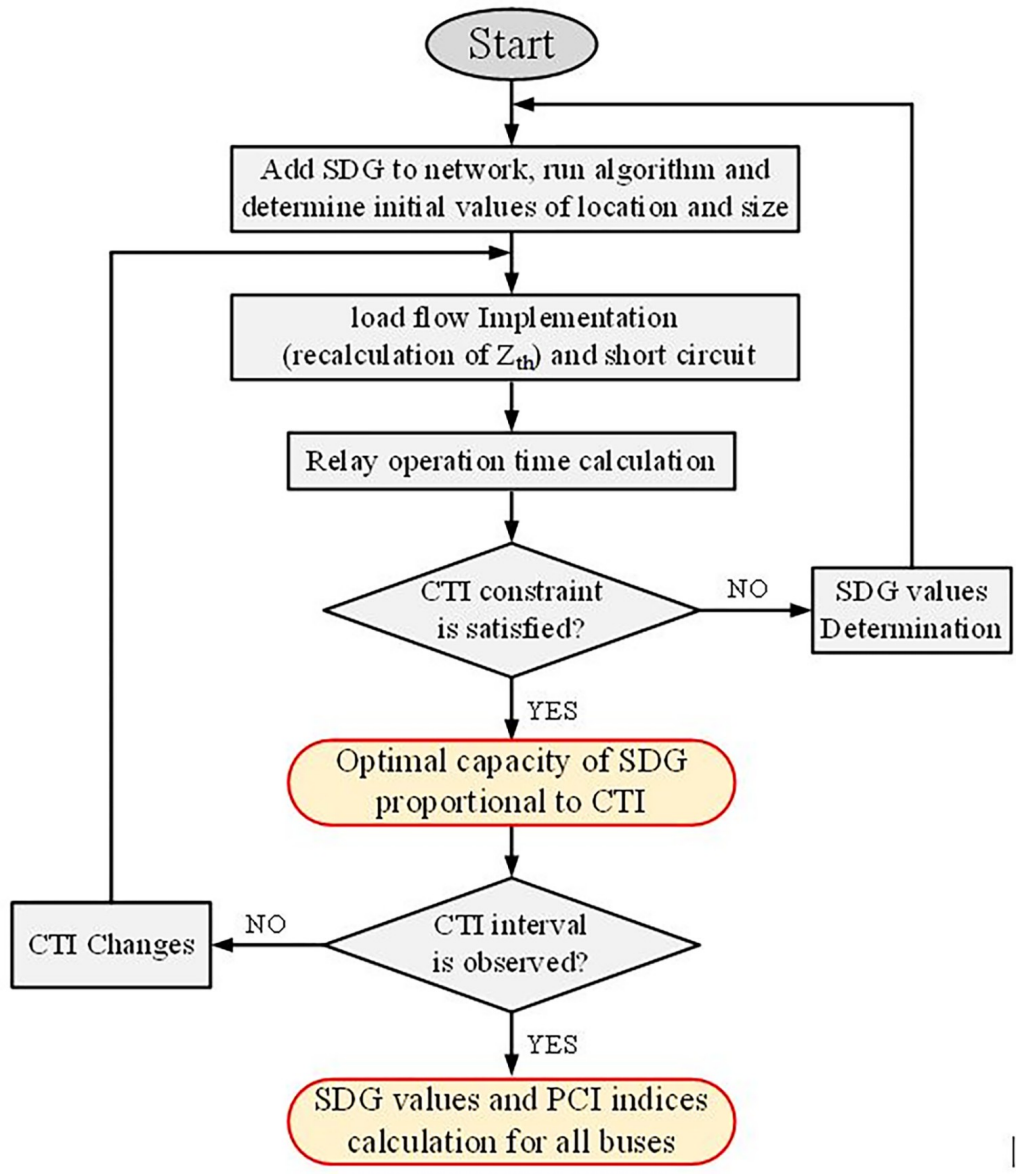
Flowchart of optimal values of SDG and penetration index of each bus.

The flowchart in Fig 2 shows the process of calculating the maximum penetration of SDG and calculating the PCI index, which aims to reach the maximum capacity of SDG in the network with different values of CTI and provide a PCI for the use of distribution network designers.

It is worth mentioning that through the presented problem, it is possible to obtain the maximum possibility of infiltration of DGs into the network without disrupting the coordination of protective equipment. In other words, this paper aims to find the maximum possibility of injecting power by DGs to each bus until the protection equipment settings do not need to be changed.

### 2.2. Optimum protection coordination

The protection coordination of overcurrent relays in the fixed structure of the network can be formulated as an optimization problem. The objective function for the optimization problem is defined according to Eq (7) [32, 35–37].

$$Minimize\;T=\sum_{f=1}^{F}\left(\sum_{i=1}^{N}t_{i,f}^{P}+\sum_{j=1}^{N}t_{j,f}^{P}\right),\;\forall i,j,f$$ (7)

where T is the operation time of all relays, F is the total number of fault locations and f is the counter of fault locations, N is the total number of relays, i and j are the relay counters, and t_i, f_^P^ and t_i,f_^B^ are primary and backup relay operation times, respectively. Time t of primary and backup relays are calculated through Eqs (8) and (9).

ti,fP=Ai,f×TDSi,f(ISCi,fIPi,f)Bi,f−1,∀i,f (8)

tj,fP=Ai,f×TDSj,f(ISCj,fIPj,f)Bi,f−1,∀j,f (9)

that A_i,f_ and A_j,f_ are the coefficients of the inverse curve type. The function B_i,f_ and B_j,f_ of the characteristic coefficients of the relay and target is minimized by fulfilling a certain number of conditions that are stated below.

The number of restrictions depends on the number of fault locations and primary and backup relays for each fault. For successful protection coordination, the backup relay is only allowed to operate at moments when the primary relay is not operating. As a result, a minimum period of time should be considered for which the backup relay gives the primary relay the opportunity to operate. This time gap is called CTI and it is expressed according to Eq (10) [32, 35–37]. The value of CTI is usually between 200 and 500 ms, which is considered equal to 311 ms in this paper.


$$t_{j,f}^{B}‐t_{i,f}^{P}\geq \mathrm{CTI},\;\forall i,j,f$$ (10)

The values of TDS, I_P_, A and B of the relay directly affect its operation time and their approximate values are also obtained as Eqs (11)–(14).


$$TDS_{\min}\leq TDS_{i,f},TDS_{j,f}\leq TDS_{\max},\;\forall i,j,f$$ (11)

$$\max(I_{P\min},I_{L\max})\leq I_{Pi,f},I_{Pj,f}\leq \min(I_{P\min},I_{L\max}),\;\forall i,j,f$$ (12)

$$A_{\min}\leq A_{i,f},A_{j,f}\leq A_{\max},\;\forall i,j,f$$ (13)

$$B_{\min}\leq B_{i,f},B_{j,f}\leq B_{\max},\;\forall i,j,f$$ (14)

The minimum and maximum range of TDS is according to the curves given in the relay. The following two points should be kept in mind in the border limits of the I_P_ clause:

- The overcurrent relay should not function for normal currents passing through network lines, that is, the maximum load current (Maximum Load Current (I_Lmax_)) should not cause the relay to operate. Therefore, the I_P_ setting factor of the relay must be greater than the maximum load current passing through the relay, taking into account the permissible overload.
- The setting should be such that it operates for the lowest possible short-circuit current (I_SCmin_) in the protection zone of the relay. Therefore, its coefficient should be lower than the minimum short circuit current in the lines that support them.

The values of load current and short circuit are obtained from the results of load distribution and short circuit [34]. The value of A and B coefficients is also considered between [0.14–80] and [0.02–2], respectively, according to the ranges available in the relay type for the characteristics curve. The operation of the relay should not be too fast or too slow in terms of time. Therefore, a condition for the time limitation of relay operation is applied to it with Eq (15).

$$t_{\min}\leq t_{i,f}^{P},t_{i,f}^{B}\leq t_{\max},\;\forall i,j,f$$ (15)

that t_min_ and t_max_ are the minimum and maximum allowed time of relay operation and equal to 0.1 to 2.5 seconds, respectively.

### 2.3. SDGS effect on operation and protection

In this section, the goal is to achieve the appropriate location and size of SDGs by considering technical, protective and cost indicators. The proposed design is presented in the framework of a four-objective optimization problem, the purpose of which is to minimize energy loss, improve the voltage security index, reduce cost, and reduce the total operation time of relays (decrease CTI deviation). Equations of load distribution, losses, voltage security, cost, and protection coordination are the limitations of the problem. The objective functions are expressed in Eqs (16)–(19).

$$\min\;AEL=\sum_{h\in H}\sum_{l\in L}R_{l,h}\times I_{l,h}^{2}$$ (16)

$$\max\;\mathrm{VSI}=\sum_{h\in H}WSI_{h}$$ (17)

$$\min\;PI=\sum_{j\in N(i)}\sum_{i\in N}\sum_{f\in F}\left(CTI_{j,i,f}-CTI_{ref}\right)$$ (18)

$$\min\;Cost=\sum_{h\in H}C_{k}S_{k}^{DG}+\sum_{h\in H}\sum_{k\in K}\left(a_{k}+b_{k}P_{k,h}^{DG}+c_{k,h}{\left(P_{k,h}^{DG}\right)}^{2}\right)$$ (19)

where AEL is the energy loss, H,h is the total number of hours and its counter, L,l is the total number of distribution network lines and its counter, R is the resistance of the distribution network lines, VSI is the voltage security index, WSI is worst security index for each hour, PI is protection index, CTI_ref_ is reference CTI value (0.3 s), Cost is total operating cost ($/year), C_k_ is SDG installation cost ($/year), S_k_^DG^ is the capacity installed in the k^th^ bus, a, b, c are coefficients of fuel cost function ($/MWh^2^) and P_DG_ is the active power produced by SDG.

Based on this, the limits of the objective function are mentioned as Eqs (20)–(32).

$$P_{k,h}^{DS}+P_{k,h}^{DG}-\sum_{m\in M}A_{k,m}^{DL}P_{k,m,h}^{DL}=P_{k,h}^{L},\;\forall k,m,h$$ (20)

$$Q_{k,h}^{DS}+Q_{k,h}^{DG}-\sum_{m\in M}A_{k,m}^{DL}Q_{k,m,h}^{DL}=Q_{k,h}^{L},\;\forall k,m,h$$ (21)

$$P_{k,m,h}^{DL}=G_{k,m}^{DL}{\left(V_{k,h}\right)}^{2}-V_{k,h}V_{m,h}\{\begin{array}{l} G_{k,m}^{DL}\;\cos(\theta_{k,h}-\theta_{m,h})+\\ +B_{k,m}^{DL}\;\sin(\theta_{k,h}-\theta_{m,h}) \end{array}\},\;\forall k,m,h$$ (22)

$$Q_{k,m,h}^{DL}=-B_{k,m}^{DL}{\left(V_{k,h}\right)}^{2}+V_{k,h}V_{m,h}\{\begin{array}{l} B_{k,m}^{DL}\cos(\theta_{k,h}-\theta_{m,h})-\\ -G_{k,m}^{DL}\sin(\theta_{k,h}-\theta_{m,h}) \end{array}\},\;\forall k,m,h$$ (23)

$$\alpha_{k,h}=0,\;\forall k,h$$ (24)

$$V_{k,h}=V_{ref},\;\forall k,h$$ (25)

$$\sqrt{{\left(P_{k,m,h}^{DL}\right)}^{2}+{\left(Q_{k,m,h}^{DL}\right)}^{2}}\leq S_{k,m}^{DL,U},\;\forall k,m,h$$ (26)

$$\sqrt{{\left(P_{k,h}^{DS}\right)}^{2}+{\left(Q_{k,h}^{DS}\right)}^{2}}\leq S_{k}^{DS,U},\;\forall k,h$$ (27)

$$V_{k}^{L}\leq V_{k,h}\leq S_{k}^{U},\;\forall k,h$$ (28)

$$\begin{array}{l} WSI_{h}={\left(V_{p-1,h}\right)}^{4}‐4{\left(V_{p-1,h}\right)}^{2}(R_{p-1,p}P_{p-1,p,h}^{DL}+X_{p-1,p}Q_{p-1,p,h}^{DL})\\ \;=4(X_{p-1,p}P_{p-1,p,h}^{DL}+R_{p-1,p}Q_{p-1,p,h}^{DL}),\;\forall h \end{array}$$ (29)

$$WSI^{L}\leq WSI_{h},\;\forall h$$ (30)

$$\sqrt{{\left(P_{k,h}^{DG}\right)}^{2}+{\left(Q_{k,h}^{DG}\right)}^{2}}\leq S_{k}^{DG},\;\forall k,h$$ (31)

$$0\leq S_{k}^{DG}\leq S_{k}^{DG,U},\;\forall k$$ (32)

where P^DS^ and Q^DS^ are the active and reactive power injected in the distribution substation, P^DG^ and Q^DG^ are the active and reactive power injected SDG, P^DL^ and Q^DL^ are the active and reactive power passing through the distribution line, P^L^ and Q^L^ are the active and reactive loads in the k^th^ bus, ADL is bus connection matrix, G^DL^ and B^DL^ distribution line conductivity and susceptance, θ_k_ and θ_m_ bus voltage angle in radians, S^DL,U^, S^DS,U^, S^DG,U^ are the respective maximum capacities, V^L^ and V^U^ are the minimum and maximum voltage of the bus and p,p-1 is the weakest bus in terms of voltage and the weak bus connected to it.

The described problem is a multi-objective optimization. First, it is necessary to extract a single-objective formula to achieve the optimal solution by ordinary solvers. For this purpose, the simulated annealing optimization technique based on the sum of weighted functions method has been used [38]. The objective function, as given in (33), is equal to the sum of the objective functions expressed in (16) to (19) with coefficients ω_AEL_, ω_VSI_, ω_PI_ and ω_Cost_.

As mentioned in (34), the sum of the coefficients must be equal to 1. Then the values of AEL, VSI, PI and Cost functions can be calculated by taking into account the operating and protection limits, respectively.


$$\min\;F=\omega_{AEL}AEL+\omega_{VSI}VSI+\omega_{PI}PI+\omega_{Cost}Cost$$ (33)

$$\omega_{AEL}+\omega_{VSI}+\omega_{PI}+\omega_{Cost}=1$$ (34)

To find the values, first, the problem is investigated in a single-objective manner to see the effect of each of the indicators on the location and size of the SDG. Then it is checked as a percentage with constant values so that condition (34) is always observed. At the end, the values of this relation and the indicators are calculated simultaneously by the algorithm. It should also be noted that the target functions of this article are non-linear and their solution requires meta-heuristic evolutionary algorithms.

## 3. Simulated Annealing Optimization Algorithm (SAOA)

Simulated Annealing Optimization Algorithm (SAOA) is a simple and effective meta-heuristic optimization algorithm in solving optimization problems in large search spaces [39]. This algorithm is mostly used when the search space is discrete. For problems where finding an approximate answer for the global optimum is more important than finding an exact answer for the local optimum in a limited and specific time, simulated refrigeration may be preferable to the rest of the methods.

To solve an optimization problem, SAOA first starts from an initial solution and then moves to neighboring solutions in an iteration loop. If the neighboring solution is better than the current solution, the algorithm places it as the current solution (moves to it), otherwise, the algorithm accepts that solution as the current solution with probability e^-ΔE/T^. In this equation, ΔE is the difference between the objective function of the current solution and the neighboring solution, and T is a parameter called temperature.

**Fig 3:**
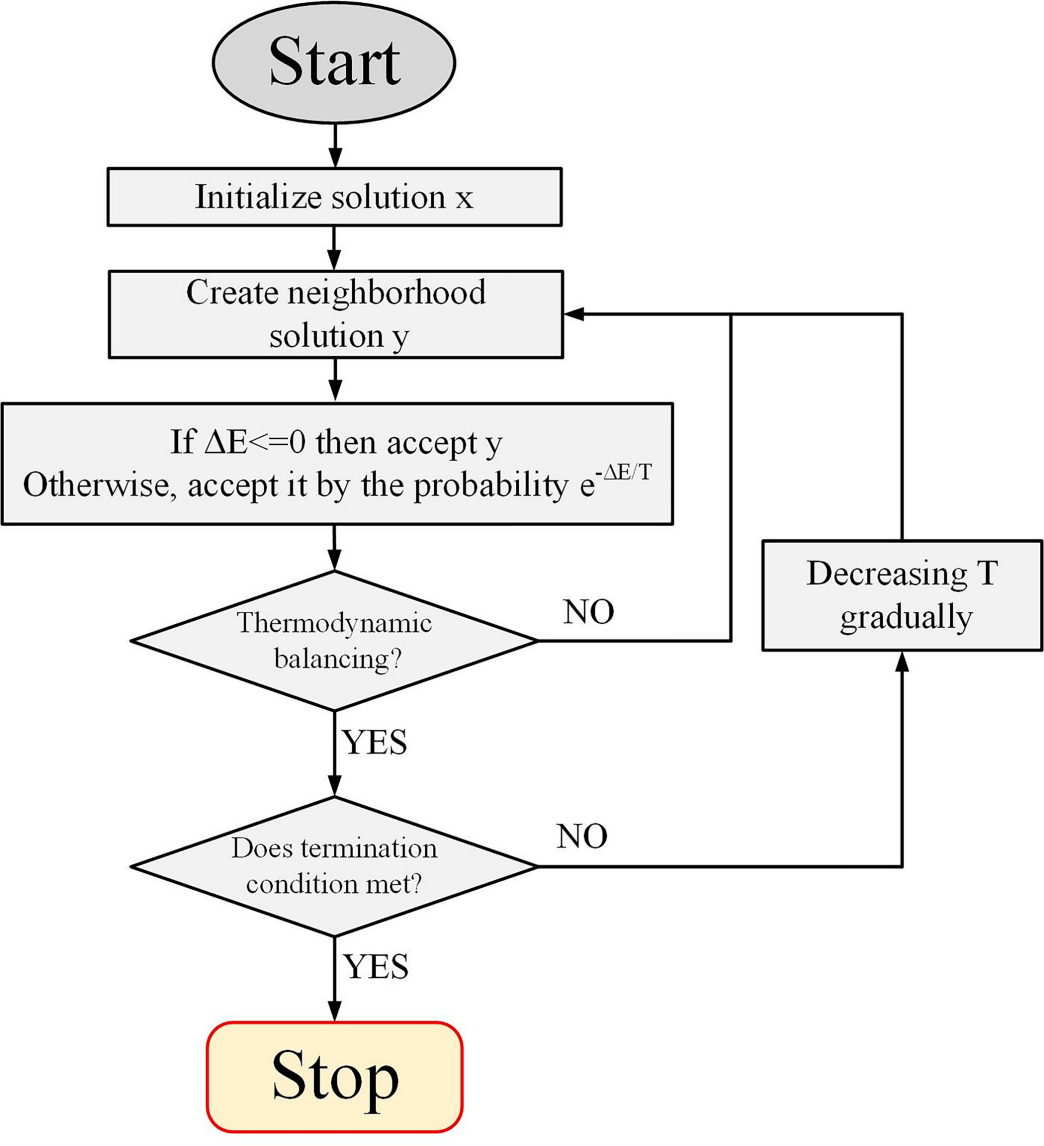
Flowchart of SAOA.

At each temperature, several iterations are run and then the temperature is slowly lowered. In the early stages, the temperature is set too high to make it more likely to accept worse solutions. As the temperature gradually decreases, in the final steps there will be less chance of accepting worse solutions and thus the algorithm will converge towards a good solution. Fig 3 shows the flowchart of this algorithm.

In the SAOA simulation algorithm, starting from an arbitrary state of the physical system, a state is reached where the internal energy of the system is minimum in that state (the system will have the lowest energy in that state). To do this, the algorithm starts from an arbitrary point and then selects a neighboring state. After that, it probabilistically decides to stay in the current state or move to the neighboring state. The sum of these possible displacements leads the system to a state with lower internal energy. This is done until the system reaches a rational state or the amount of calculations exceeds a certain threshold.

Currently, MGs have a large search space for optimization due to the diversity of resources and complexity in energy production and consumption. SAOA optimization algorithm can more fully explore the search space and find better solutions for optimal exploitation and conservation due to its ability to exit from local optima. Also, in MGs, there are uncertainties and sudden changes in production and consumption due to renewable resources such as wind and sun that have natural fluctuations. The SAOA optimization algorithm can deal well with these instabilities and perform optimization in the presence of these fluctuations.

Changing the parameters of the SAOA optimization algorithm will have a direct effect on the coordination of protection and optimal operation in MGs with distributed generation. For optimal protection and operation, choosing an appropriate initial temperature (not too high or too low) helps the algorithm explore the search space better, especially in cases where MGs face scattered generation fluctuations such as solar and wind power.

Also, in MGs where there are large fluctuations due to rapid changes in energy production from distributed sources such as wind or solar, slow temperature reduction can help to find better solutions for coordinating protection and optimal operation. However a balance must be established between the speed of temperature reduction and the calculation time.

For MGs, increasing the number of iterations at each temperature allows the algorithm to find better solutions, especially in situations where there are instabilities in distributed generation. This work can lead to improved protection and optimal operation of the system.

As a result, changing the parameters of the simulated refrigeration algorithm has a profound effect on the quality of results and execution time in the problems of coordination of protection and optimal operation in MGs. Appropriate settings of initial temperature, rate of temperature decrease, number of iterations and probability of accepting worse solutions help the algorithm reach more stable, optimal and less expensive solutions.

## 4. Numerical results and discussion

In this paper, a network of 30 buses and 69 buses has been used to perform calculations. To calculate the short circuit current and as a result calculate the I_P_ and TDS values, the fault resistance is omitted. The resources available in the network as well as DGs are of synchronous and inverter based type. In addition, it is shown in this paper that higher values of PCI mean more possibility for DGs to penetrate the network.

### 4.1. Maximum penetration capacity results

In this part, the goal is to find the maximum network penetration capacity in such a way that protection coordination can be established only by changing the CTI. Table 1 shows the PCI values in each bus.

**Table 1:** Comparing the values of PCI in the distribution section of the 30 bus network with [32].

Bus connected to SDG	[32]	[32]	Proposed method
**2**	50.64	64.82	82.15
**4**	18.06	23.12	27.57
**6**	16.91	21.64	26.08
**8**	25.31	23.40	29.68
**10**	19.15	24.97	31.27
**12**	22.57	28.89	35.74

**Fig 4:**
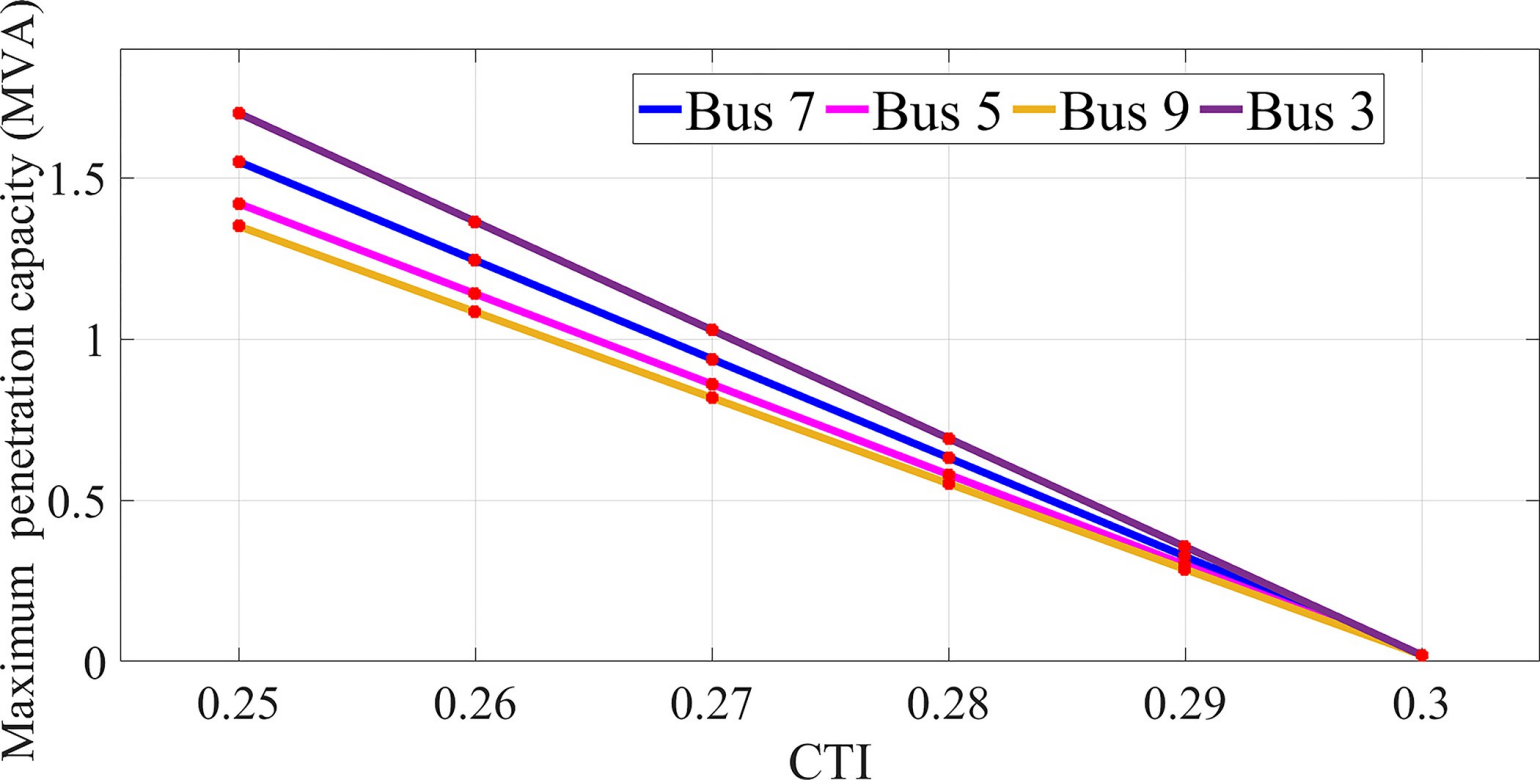
Changes of the maximum SDG penetration capacity in each CTI bus in 30-bus network.

Fig 4 shows the maximum value of SDG penetration in other tires for different CTI values. The maximum penetration value in this network is equal to 14.5 MVA.

As can be seen, the maximum capacity that can be installed in bus 2 is possible. Also, according to the impedance matrix of the network, it can be seen that the best bus for placing SDG is Bus No. 2, which has the lowest Z_th_ value.

### 4.2. DG effect on operation results

In this section, the effect of the DG on the operation and protection of the network will be investigated. These results are analyzed in two networks of 30 and 69 bases system.

#### 4.2.1. 30 buses test system results

Based on the results of load distribution with Newton-Raphson method, without considering SDGs, bus 14 has the lowest voltage value compared to other buses. The maximum loss at full load is equal to 1.392 MW. In this network, resources with a maximum capacity of 3 MVA can be installed in all buses. Table 2 shows the exploitation and protection indices for fixed weight values of 0.25, 0.33 and 1.

**Table 2:** The values of operation and protection indices considering the fixed weight value.

ω_AEL_	ω_VSI_	ω_PI_	ω_Cost_	AEL (MWh)	VSI	PI (s)	Cost (M$/year)
1	0	0	0	12.347	22.43	3.05	21.75
0	1	0	0	13.472	23.87	2.94	19.10
0	0	1	0	15.247	22.18	2.24	16.43
0	0	0	1	18.075	22.31	2.56	15.25
0.5	0.5	0	0	12.942	22.97	2.61	18.24
0.5	0	0.5	0	13.347	22.58	2.50	17.45
0.5	0	0	0.5	13.732	22.87	2.72	17.74
0	0.5	0.5	0	14.284	23.38	2.41	16.92
0	0.5	0	0.5	14.392	23.52	2.78	18.42
0	0	0.5	0.5	14.827	22.84	2.41	16.94
0.34	0.33	0.33	0	13.437	23.61	2.40	18.55
0.33	0.34	0	0.33	13.524	22.54	2.78	17.95
0.33	0	0.34	0.33	13.670	22.62	2.58	18.12
0	0.33	0.33	0.34	14.045	22.76	2.43	17.45
0.25	0.25	0.25	0.25	13.930	22.43	2.46	17.79

According to the results of lines 2–5 in Table 2:

- Minimum and maximum values of energy losses equal to 12.347 and 18.075 MWh,
- Voltage stability index 20.43 and 22.85,
- Protection index 2.24 and 3.05 seconds,
- And finally, for the cost function, it is equal to 15.25 and 21.75 million dollars per year.

According to the results, the changes of functions do not overlap enough; For example,

- The reduction of energy losses (approximately 45.5%) is equal to the increase of the installation cost.
- Increasing the amount of SDG installation in the network increases the protection index, which means the loss of protection coordination.
- Since different values have been obtained for different weight coefficients, it is necessary to determine the best solution for these functions through optimization.

Table 3 shows the results for different algorithms.

**Table 3:** The values of operation and protection indices obtained from optimization.

Algorithm	ω_AEL_	ω_VSI_	ω_PI_	ω_Cost_	AEL (MWh)	VSI	PI (s)	Cost (M$/year)
SAOA	0.11	0.27	0.48	0.14	13.743	23.72	2.38	17.06
CSA	0.12	0.24	0.49	0.15	14.037	23.35	2.42	17.43
PSO	0.14	0.27	0.45	0.14	14.178	22.94	2.47	18.45

According to the results, SAOA has obtained the best values for the indicators by being placed in the optimal points. The weight coefficients for ω_AEL_, ω_VSI_, ω_PI_ and ω_Cost_ are 0.11, 0.27, 0.48 and 0.14, respectively. Also, the impact of SDG installation location and capacity on exploitation and protection indicators is shown in Table 4 by SAOA results.

**Table 4:** The SDG location and capacity effect on operation and protection indicators in 30 buses system.

Maximum installable capacity (MVA)	0	1.5	3
Installation location (Bus)	-	2–10, 12–14	2–5, 7–8, 1, 12–14
Installed capacity (MVA)	0	1.34, 1.11, 1.07, 1.13, 0.9, 1.17, 1.23, 0.93, 1.22, 1.12, 0.72, 0.64	2.94, 1.67, 1.35, 1.38, 1.52, 1.48, 1.55, 1.74, 0.83
Total installed capacity (MVA)	0	12.58	14.46
Cost (M$/year)	0	15.63	17.06
Energy losses (MWh)	20.713	15.472	13.743
Maximum voltage drop (pu)	0.032	0.017	0.011
VSI	21.93	23.47	23.72
PI (s)	0	1.89	2.38
Maximum ΔCTI (s)	0	0.05	0.05
PCI (MVA/s)	0	251.6	289.2

So, after optimization,

- The energy loss has decreased by 33.6% compared to the initial value and the voltage security index has improved by 11%.
- The voltage drop has been upgraded from 0.968 to 0.989 PU.
- The protection index reached from the critical value of 3.05 s to the value of 2.38 s, which means full compliance with the protection coordination restrictions.
- Increasing the number of installed SDGs increases the cost of installation, reduces losses and improves voltage security and disrupts protection coordination.
- The higher the installation capacity, the less the possibility of placing on the buses. For example, buses 1 and 11 are not suitable places for installation. This state is shown in Table 1 and Fig 4.

Although values smaller than 3 MW can be installed on most buses, for this purpose, a minimum value for penetrating capacity and maintaining protection coordination with CTI is considered, which should not be less than 0.25 s.

The advantage of considering CTI clause is in terms of cost. Changing the CTI does not involve any cost, but changing the settings of the relays by adding a new source will be costly. In addition, if a new source is added again, the settings must be changed again, so it is better to limit the penetration capacity of SDG with CTI clause.

#### 4.2.2. 69 buses test system results

In this section, the proposed plan has been implemented on the standard network of 69 radial buses. This network is 12.66 kV and is connected to the upstream network through bus 1 and transformer. Maximum load data and line specifications are in [40]. Based on the results of load distribution using the forward-reverse method, Bus 65 has the lowest voltage value at 9:00 p.m. at the rate of 0.9102 PU and network losses of 224.95 kW. The maximum allowed value of SDGs installation is equal to 1 MVA. Other specifications such as load, minimum and maximum voltage values are the same as in the previous section.

**Fig 5:**
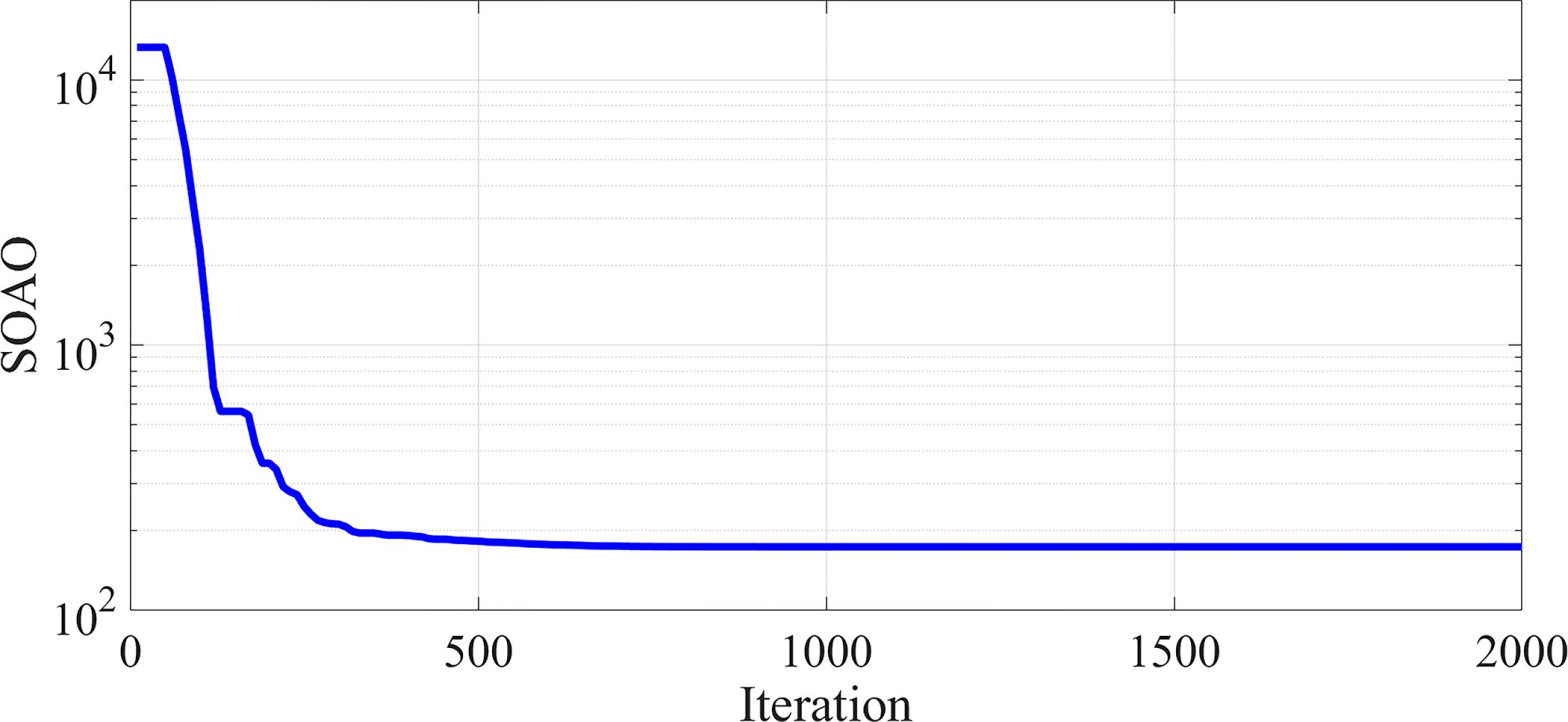
The convergence diagram of the coefficients of the objective function for the capacity of 1 MW.

Considering that the SAOA algorithm had the best optimal solutions for the previous part, the simulation results are based on this algorithm. The convergence diagram is shown in Fig 5. The weight coefficients of ω_AEL_, ω_VSI_, ω_PI_ and ω_Cost_ indicators are equal to 0.14, 0.25, 0.43 and 0.18, respectively.

Table 5 shows the indicators of exploitation and protection due to the different effect of location and capacity of SDGs. As can be seen, increasing the maximum installation capacity improves the loss indices and voltage security, but increases the installation cost and protection index.

**Table 5:** The SDG location and capacity effect on operation and protection indicators in 69 buses system.

Maximum installable capacity (MVA)	0	0.5	1
Installation location (Bus)	-	6, 7, 21, 25, 55, 58, 61, 63	7, 12, 21, 27, 57, 61, 63
Installed capacity (MVA)	0	0.4, 0.42, 0.37, 0.41, 0.4, 0.33, 0.5, 0.43	0.75, 0.4, 0.41, 0.32, 0.5, 0.66, 0.56
Total installed capacity (MVA)	0	3.26	3.47
Cost (M$/year)	0	1.97	2.08
Energy losses (MWh)	3.347	2.417	2.162
Maximum voltage drop (pu)	0.0898	0.0296	0.0175
VSI	18.73	22.37	22.45
PI (s)	0	4.53	4.71
Maximum ΔCTI (s)	0	0.05	0.05
PCI (MVA/s)	0	65.2	69.4

According to the results, the value of the PI index for increasing the capacity from 0.5 to 1 MW has reached the value of 69.4, but still the value of ΔCTI remains within the range of 0.05 s, which indicates that the protection coordination is not disturbed.

**Fig 6:**
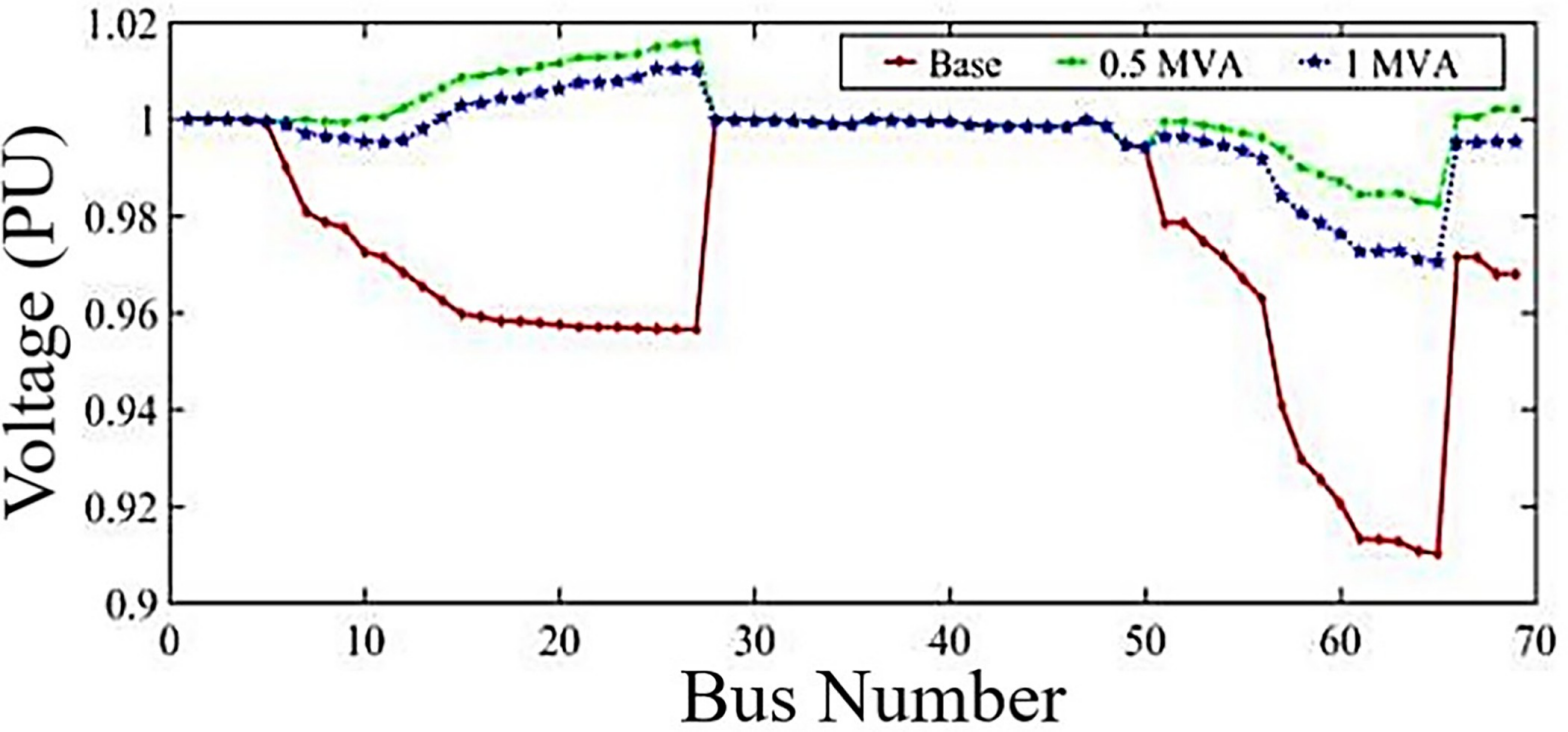
Voltage profile of 69 buses system in different modes SDG install in fully loading of the network.

In the final optimal state, the total installed capacity is equal to 3.47, which has led to a reduction in losses of about 35.4%, a reduction in the maximum voltage drop from 0.9102 PU to 0.9825 PU, an improvement in the voltage security index from 18.74 to 22.45. Fig 6 shows the voltage profile for each mode of SDG installation in the grid after optimization.

## 5. Conclusion

In this paper, the design of adaptive protection coordination and optimal parameters of numerical overcurrent relays in the distribution network was investigated considering the influence of inverter based DG sources. Therefore, the role and effect of installing DGs in the distribution network was raised, one of the most important problems of which is the effect on protection coordination. In addition, by using the PCI index, the maximum penetration rate of DGs in each bus was obtained so that the protection coordination created is not disturbed and is only limited by the CTI constraint. Also, using this index, Z_th_ of the distribution network and SDG impedance, an idea of determining the location and capacity of SDGs was proposed, which was implemented and investigated in two sample distribution networks. Finally, the effect of operational indicators such as losses, voltage, cost and protection index in determining the optimal location and capacity of resources was discussed. The result of this issue was that to place resources in the distribution network, it is not enough to consider only the loss index, voltage, cost or protection coordination, and the indices should be considered together to reach a suitable solution.

## Supporting information

S1 File (RAR)

## Abbreviation list

DG: Distributed Generation
MG: Micro-Grid
FCL: Fault Current Limiters
DS/DOCR: Dual Setting Directional Overcurrent Relay
BEL: Battery Electric Locomotives
SMINLP: mixed- integer nonlinear stochastic programming
JPC: joint probabilistic constraints
PV: Photovoltaic
MMG: Multiple MG
SDG: Synchronous DG
ADN: Active Distribution Network
SAOA: Simulated Annealing Optimization Algorithm
TDS: Time-Dial Sitting
PCI: Protection Coordination Index
CTI: Coordination Time Interval
AEL: Annual Energy Loss
VSI: voltage security index
WSI: worst security index
